# Local use of dexamethasone in the treatment of ocular myasthenia gravis

**DOI:** 10.1186/s12886-020-01697-2

**Published:** 2020-10-28

**Authors:** Minghua Shi, Yingjia Ye, Junping Zhou, Aijiao Qin, Jing Cheng, Hongxing Ren

**Affiliations:** Department of Strabismus and Pediatric Ophthalmology, Wuhan Aier Eye Hospital (Hanyang), Wuhan, 430020 China

**Keywords:** Ocular myasthenia gravis, Strabismus, Corticosteroids, Dexamethasone, Local injection

## Abstract

**Background:**

At present, patients with ocular myasthenia gravis (OMG) are typically treated with systemic drugs. We investigated the use of dexamethasone injected in the peribulbar region or extraocular muscle to treat patients with OMG.

**Methods:**

Patients with OMG were given dexamethasone via peribulbar injection or direct injection into the main paralyzed extraocular muscles, once a week, for 4–6 weeks. The severity of diplopia, blepharoptosis, eye position, and eye movement were evaluated before and after treatment. The duration of follow-up time was ≥6 months.

**Results:**

Among the 14 patients with OMG who received this treatment, mean age was 38.7 ± 29.7 years. After treatment, symptoms were relieved in 12 patients (85.7%), 1 patient (7.1%) had partial response to treatment, and 1 patient (7.1%) had no response. Two patients (14.2%) experienced symptom recurrence during the follow-up period.

**Conclusions:**

Dexamethasone peribulbar or extraocular muscle injection is effective in the treatment of patients with OMG and may replace systemic drug therapy.

**Trial registration:**

Chinese Clinical Trial Registry, ChiCTR2000038863, October 7, 2020.Retrospectively registered.

**Supplementary information:**

The online version contains supplementary material available at 10.1186/s12886-020-01697-2.

## Background

Myasthenia gravis (MG) is an autoimmune disease that mainly involves acetylcholine receptors (AchR) on the postsynaptic membrane at the neuromuscular junction. The annual incidence of MG is reported to range from 3 to 30 per million per year, with prevalence of approximately 150–300 per million [1–3]. About 60% MG known as simple OMG [3–5]. This proportion is higher among children, reaching 71 to 93% [5, 6].

In the past, even patients with OMG in whom symptoms were limited to the eyes were treated with systemic drugs, including acetylcholinesterase inhibitors, corticosteroids, immunosuppressants, immunomodulating agents and, recently, vascular growth factor inhibitors [7–9]. Among these treatments, oral acetylcholinesterase inhibitors and corticosteroids were used most commonly [7–9]. Few patients underwent thymectomy or other types of surgery [10]. But any type of systemic medication is likely to induce adverse reactions.

Ophthalmologists seeking to treat autoimmune ocular diseases used various methods for drug delivery, including subconjunctival, hemispheric, and retrobulbar injections, as well as intravitreal administration, and achieved good therapeutic effects. These diseases include simple ocular inflammation and ocular inflammation caused by systemic disease [11]. Local treatment with topical corticosteroids has been recommended as first-line treatment for juvenile idiopathic arthritis (JIA)-associated uveitis, when the disease is mild and patients can be weaned relatively quickly off of steroid therapy [12, 13]. The topical injection of hormone drugs such as triamcinolone acetonide or dexamethasone also has been used successfully for the treatment of thyroid-related ophthalmopathy [14, 15]. Alkawas et al. compared the efficacy of peribulbar triamcinolone acetonide to oral prednisone in the treatment of thyroid-associated ophthalmopathy. In the group treated orally, the mean exophthalmometry measurement decreased from 23 ± 1.86 mm before treatment to 19.08 ± 1.16 mm after treatment. However, in the group that received peribulbar injections, mean exophthalmometry values before and after treatment were 22.6 ± 1.98 mm and 18.6 ± 0.996 mm, respectively. Furthermore, compared with oral hormone treatment, local injections were associated with fewer side effects.

Based on these published findings, we speculate that local medication for OMG may effectively treat ocular symptoms, while decreasing the risk for side effects, compared with the use of systemic medication. This article explores the use of locally injected dexamethasone, a corticosteroid, for the treatment of patients with OMG.

## Methods

Patients who came to our hospital during the period from August 2016 to May 2019 were diagnosed with OMG based on the symptoms presented and the results of the neostigmine test. If the neostigmine test was negative, a positive results on thymic computed tomography (CT), electromyography, or the acetylcholine receptor antibody(AChR-Ab)test was required. The exclusion criteria were systemic symptoms (e.g., muscular weakness below the neck), refusal to cooperate with the treatment plan, and compliance with follow-up. This study adhered to the Declaration of Helsinki, and was approved by our hospital Ethics Committee.

A detailed medical history was obtained for each patient. All patients underwent general and ocular examinations. The general examination included routine blood and urine tests, as well as measurements of blood sugar, blood lipid, liver and kidney function, thyroid function, erythrocyte sedimentation rate, rheumatoid factor, and C-reactive protein. Each patient also underwent slit-lamp, fundus, and synoptophore examinations, as well as measurements of intraocular pressure and visual acuity. A four-hole lamp was used to check for diplopia, and the prism alternative cover test was performed to determine the degree of deviation. Blepharoptosis was classified as mild (upper eyelid margin covering < 1/2 pupils with the patient looking straight ahead), severe (completely unable to open the eyelid fissure), or moderate (between “mild” and “severe”). Ocular motor duction was graded on a scale ranging from 0 to − 5 (0, normal; 5, lack of muscle function; − 1 to − 4, not reaching the midline, in 25% increments).

Before formal treatment was initiated, all patients and their families were given detailed information about their condition and various treatment methods, including the advantages and disadvantages of each. After an understanding of the risks involved was achieved, all patients or their parents signed an written informed consent form approved by the institutional Review Board. We also obtained explicit written informed consent to publish all data related to the study (including individual details, images, and videos) from the patients or their parents (for children < 18 years of age).

All patients received local injections of dexamethasone in the peribulbar region or extraocular muscle, once a week, for 4–6 times. The site of injection was determined based on the patient’s ocular signs. In the case of a clear limitation of ocular duction, dexamethasone was directly injected into the main paralyzed extraocular muscle. If the main symptom was ptosis, with no limitation of ocular duction, a peribulbar injection of dexamethasone was administered to the inferolateral orbital quadrant. Patients were closely observed with regard to ocular position and movement, diplopia and degree of blepharoptosis, visual acuity, intraocular pressure and general condition. If the symptoms did not improve after two injections, if the patient’s condition worsened at any time, or if any sign of GMG (any muscle weakness below the neck or in the facial muscles except the orbicularis oculi) appeared, the treatment plan was discontinued, and the patient was transferred to the neurology department. After treatment, the patients were followed up monthly for 6 months, or at any sign of disease recurrence.

### Extraocular muscle injection

Patients assigned to the extraocular muscle injections group received 0.5% dexamethasone (0.2 mL) and 2% lidocaine (0.2 mL). After topical anesthesia with proparacaine, the clinician disinfected the skin around the eye and the conjunctival surface with 5% povidone iodine. The eyelid was opened, and the paralyzed extraocular muscle was clamped with toothed forceps. Injections were administered with 30G needles, which were inserted at the point of extraocular muscle attachment, then advanced 8–10 mm along the muscle to inject dexamethasone directly into the muscle sheath.

### Peribulbar injection

Patients assigned to the peribulbar injection group received 0.5% dexamethasone (0.5 mL), which was administered with an 27G needle. The drug was slowly injected inside the orbital rim of the inferolateral quadrant, posterior to the orbital septum.

### Statistical analysis

Statistical analysis was performed with SPSS statistical software, version 17.0. The degree of strabismus was compared before vs. after treatment with the t-test. The relationship between the degree of ocular duction limitation and recovery time was tested with the bilateral Pearson correlation test.

## Results

During the period from August 2016 to May 2019, 20 patients at our hospital were diagnosed with MG. Sixteen patients had OMG, and 4 patients had mild general myasthenia gravis (GMG). Four patients with GMG were transferred to the external neurology department. Two patients with OMG did not consent to receive treatment. The remaining 14 cases of OMG received the treatment regimen, including a 8-years old boy, who was successfully treated by local injection under surface anesthesia. Among them, 2 patients had an irregular history of medical treatment. Patient age ranged from 8 to 92 years (average, 38.7 ± 29.7 years). Average best-corrected visual acuity (BCVA; log MAR) was 0.11 ± 0.03 in the right eyes and 0.14 ± 0.10 in the left eyes.

All 14 patients had strabismus, diplopia, and ptosis. Four patients had headache, and neck weakness. The neostigmine test yielded positive test results in12 cases. CT scans revealed thymic hyperplasia in 3 cases and AChR- Ab positive disease in 6 cases. See Table 1 for details.

**Table 1:** General patient information

Patient number	1	2	3	4	5	6	7	8	9	10	11	12	13	14	Average
BCVA															
od	0.1	0.1	0.1	0.1	0.1	0.1	0.2	0.1	0.1	0.1	0.1	0.1	0.2	0.1	0.11 ± 0.03
os	0.1	0.1	0.1	0.1	0.1	0.1	0.4	0.1	0.1	0.1	0.1	0.1	0.4	0.1	0.14 ± 0.10
Duration	8	12	3	3	3	6	1	2	4	6	3	3	3	4	4.35 ± 2.84
Neostigmine test	+	+	+	+	+	+	+	+	–	+	–	+	+	+	
Thymus CT	–	–	–	–	+	–	–	–	+	+	–	–	–	–	
AchR-Ab	–	+	–	+	–	–	+	–	+	+	–	–	–	+	
Systemic disease	No	No	No	No	No	No	No	No	No	No	No	No	No	No	
History of treatment	No	No	No	Yes	No	No	No	No	No	No	No	No	No	Yes	
Ptosis	Bilateral	Left	Right	Bilateral	Right	Left	Bilateral	Right	Bilateral	Right	Left	Right	Right	Bilateral	
Diplopia	+	+	+	+	+	+	+	+	+	+	+	+	+	+	
Ocular Duction Limitation	+	+	+	–	+	+	+	+	_	+	+	+	–	–	
Deviation before treatment (PD)															
horizontal^a^	−40	−30	−30	−20	−60	−30	− 30	−40.	−20	−40.	−25	−35	−20	+ 20	−28.5 ± 11.1
vertical	2	3	0	0	8	10	20	6	3	5	12	10	0	3	5.85 ± 5.68
Injection site	RMR	LMR	RMR	BMR	RMR	LMR	LMR + LSR	RMR	BMR	RMR	LMR	RMR	RP	RLR	
Injection times	6	4	4	4	6	4	2	4	4	5	4	4	4	4	4.23 ± 1.01
Deviation after treatment (PD)															
horizontal^a^	−5	−3	−3	0	−3	−5	− 30	−5	−3	− 3	−4	0	− 2	+ 3	−4.92 ± 7.38
vertical	0	2	2	3	0	5	10	0	0	0	5	2	0	0	2.07 ± 2.92
Follow up time(months)	25	23	23	21	19	19	–	17	16	13	10	9	8	6	16.0 ± 6.33
Recurrence	NO	NO	NO	NO	NO	NO	–	NO	Yes	NO	NO	NO	NO	Yes	

Duration: time from symptom onset to presentation at our institution; *AChR Ab* acetylcholine receptor antibody; *BCVA* best-corrected visual acuity; *OD* right eye; *OS* left eye; *PD* prism diopters; *RMR* right medial rectus muscle: *LMR* left medial rectus muscle; *BMR* bilateral medial rectus muscles; *LSR* left superior rectus muscle; *RP* right peribulbar area; *RLR* right lateral rectus muscle

^a^Average horizontal deviation before and after treatment (absolute value)

Among 14 patients, 3 cases had normal ocular duction; 7 cases had adduction; 1 case had abduction; 3 cases had adduction and supraduction. The information related to each patient’s ocular characteristics and the site of injection are detailed in Table 1 and summarized in Supplementary file 1.

### Treatment outcomes

Patient 7, a 92-year-old female, did not significantly improve after receiving 2 injections in the area adjacent to the medial and superior rectus muscles. She was transferred to the neurology department. The exotropia and diplopia experienced by Patient 13, a 73-year-old female, resolved after treatment, but her blepharoptosis improved only slightly. The remaining 12 patients achieved good treatment results. Patient 4 and Patient 9 had intermittent exotropia without obvious limitations in duction; their symptoms resolved after a single injection. The other 9 patients with limited duction presented with a resolution of their ptosis, neck weakness, and headache symptoms after one-time treatment. Diplopia symptoms disappeared in all patients after 4 injections. However, the complete recovery of extraocular muscle strength was delayed in some patients. Patients 1, 5, and 10 received another 1–2 injections because persistent limitations in ocular duction. After 3 months of follow-up, ocular duction had returned to normal in these patients. Among the patients who ultimately recovered from ptosis, the time to recovery time was ≤1 week. The average time to recovery from diplopia was 1.75 ± 0.62 weeks; the average time to recovery of ocular duction was 4.55 ± 2.44 weeks. The time to recovery of ocular duction was inversely correlated with the severity of the limitation (*R* = 0.13, *P* = 0.739). The severity of ocular symptoms and recovery time are shown in Table 2.

**Table 2:** Symptom severity and recovery time

Patient no.	Symptom severity		Recovery time (weeks)		
	Ptosis (Side: Degree)	Ocular duction (Eye: Duction, Degree)	Ptosis	Diplopia	Ocular duction
1	Right: Moderate; Left: Mild	OD, Adduction, − 3	1	3	8
2	Left: Moderate	OS: Adduction, −1	1	2	4
3	Right: Moderate	OD: Adduction, −2	1	2	3
4	Bilateral: Mild	Normal	1	1	__
5	Right: Moderate	OD: Adduction, −3	1	2	8
6	Left: Moderate	OS: Adduction, − 3 mm; Supraduction, −1	1	2	4
7	Right: Mild; Left: Severe	OS: Adduction, −1; Supraduction, −2	No improvement	No improvement	No improvement
8	Right: Mild	OD: Adduction, −1	1	1	2
9	Bilateral: Mild	Normal	1	1	–
10	Right: Moderate	OD: Adduction, −3	1	2	6
11	Left: Mild	OS: Adduction, −2; Supraduction, −1	1	2	3
12	Right: Moderate	OD: Adduction, −2	1	2	3
13	Right: Severe	Normal	Improvement	1	–
14	Bilateral: Moderate	OD: Abduction, −3	1	3	5

*OD* right eye; *OS* left eye

Blepharoptosis was classified as mild (upper eyelid margin covering < 1/2 pupils with the patient looking straight ahead), severe (completely unable to open the eyelid fissure), or moderate (between “mild” and “severe”). Ocular motor duction was graded on a scale ranging from 0 to −5 (0, normal; 5, lack of muscle function; −1 to −4, not reaching the midline, in 25% increments

### Recurrence

One case (Patient 9) relapsed 1 year after recovery. The symptoms were alleviated after 4 local injections of dexamethasone. The condition is stable at present. A cured esotropia patient (Patients 14) developed exotropia and diplopia 6 weeks after withdrawal. After 4 injections of dexamethasone into the medial rectus muscles of both eyes, the symptoms were relieved once more. However, the patient’s symptoms recurred again after 5 weeks. We did not believe that this patient was able to achieve drug-independent remission, so local injections were administered once per month. At present, the patient’s symptoms are well controlled.

In short, among the 14 cases, 11 cases achieved drug-independent remission; 1 case achieved drug-dependent remission; 1 case improved; 1 case failed to improve. The overall cure rate was 85.7%. No patient converted to GMG. The average horizontal deviation (absolute value) was 28.57 ± 17.5 PD before treatment and 4.92 ± 7.38PD at the time of the last follow-up (t = − 4.73, *P* < 0.001). The average vertical deviation decreased from 5.85 ± 5.68 PD before treatment to 2.07 ± 2.92 PD after treatment (t = − 3.02, *P* = 0.003). For details see Table 1.

### Complications

All patients had no local or systemic complications except subconjunctival hemorrhage caused by injection. Including decreased vision, increased peribulbar pressure, conjunctival and corneal lysis, obesity and so on.

### Representative cases

#### Case 1

**Fig. 1:**
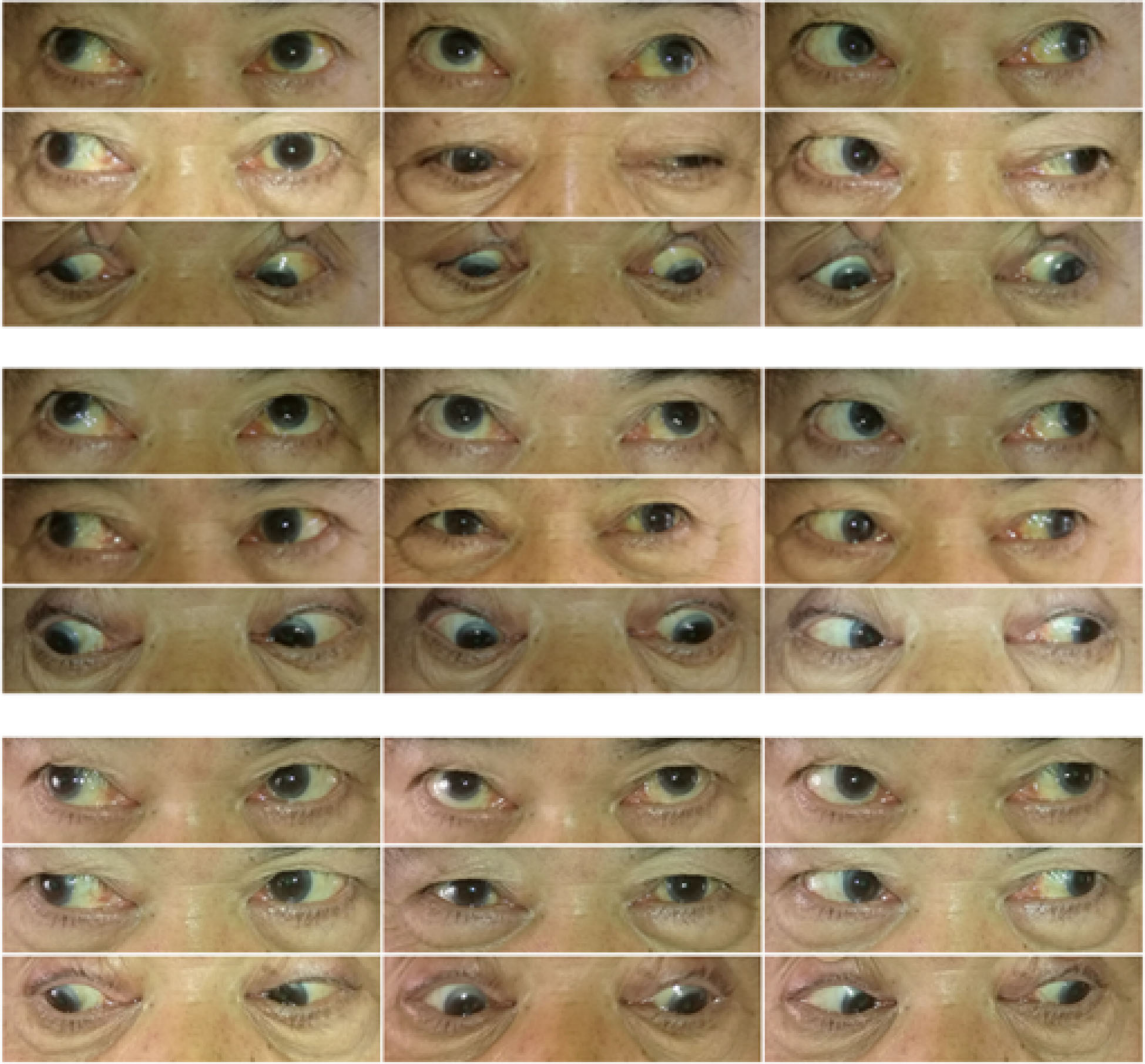
Eye position in Patient 6. The photographs of eye position from top to bottom: before treatment,1 week, 1 month after treatment

Patient 6 was a 73-year-old male patient who had experienced diplopia and blepharoptosis for 1 month, with no headache, neck weakness, or other symptoms. Upon physical examination, the main signs were left eyelid droop and large-angle exotropia, accompanied by a mild limitation in supraduction. Dexamethasone was injected into the left medial rectus muscle. The patient’s blepharoptosis resolved within 1 week; diplopia resolved within 2 weeks; eye movement returned to normal in 4 weeks. No recurrence was noted during the follow-up period. Photographs of this case (before treatment and 1 week and 1 month after treatment, respectively) are shown in Fig. 1.

#### Case 2

**Fig. 2:**
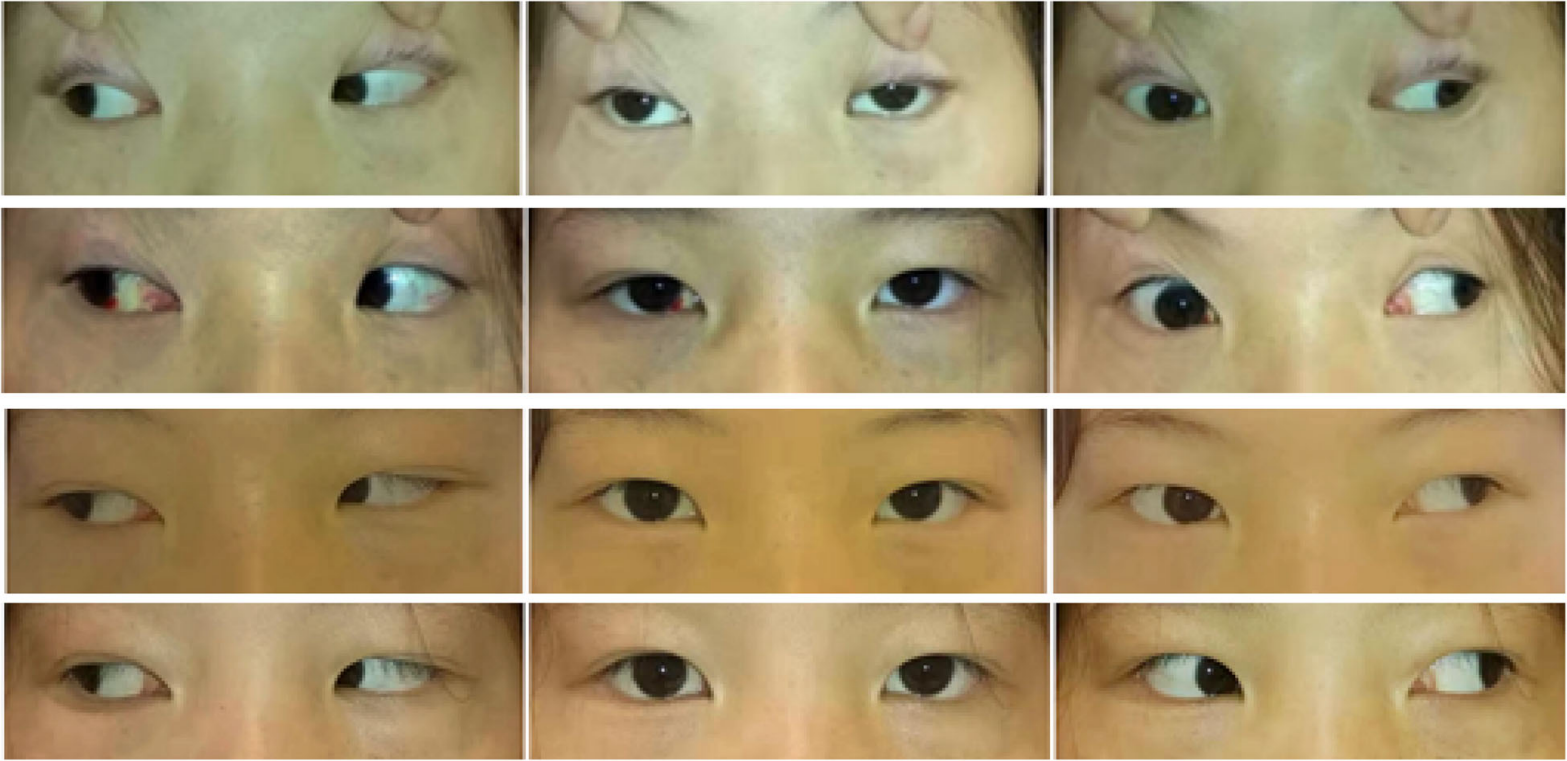
Eye position in patient 1. The photographs of eye position from top to bottom: before treatment, 1 week, 1 month, and 3 months after treatment

Patient 1 was a 14-year-old female who presented with 8-month history of blepharoptosis and diplopia, accompanied by neck weakness, headache, and inability to sustain visual focus. Main signs: moderate blepharoptosis in the right eye, mild blepharoptosis in the left eye; exotropia (right eye adduction with inability to cross the midline). Dexamethasone was injected into the right medial rectus muscle. One week later, the patient’s ptosis, headache, and neck weakness had resolved. After 3 weeks, the diplopia disappeared, and eye position was orthophoria. After 4 weeks, rotation of the right eye remained insufficient. Dexamethasone was injected another two times. After 2 months, eye movement had returned to normal, with no recurrence of symptoms over 2 years of follow-up. Photographs of this case (before treatment and 1 week, 1 month, and 3 months after treatment) are shown in Fig. 2.

## Discussion

A review of the medical literature did not reveal any reports about the local used of medication in the treatment of OMG. Whether uveitis is caused by systemic disease or thyroid-related ophthalmopathy, local inflammation is a contributing factor. Local inflammation may respond to anti-inflammatory medication. Notably, myasthenia is not the direct result of inflammation but, rather, an antibody-mediated weakness. The symptoms associated with myasthenia are caused by antibody production that is not necessarily localized, so local injections of corticosteroid are not expected to reduce the production of circulating antibodies to the acetylcholine receptor. This may be the main reason why few studies published to date have investigated the administration of local treatment for MG. However, Agius maintain that OMG may be associated with ocular muscle antigen structure and/or reduced susceptibility to risk factors [16]. Soltys et al. [17] reported that, in autoimmune MG, the neuromuscular junctions in extraocular muscles are more susceptible to complement-mediated injury than are those of other muscles. Local factors thus appear to be a major cause of OMG. Although numerous issues remain to be elucidated, the local injection of dexamethasone appears to be an effective treatment option. The local use of dexamethasone is simple, convenient, and almost risk free.

More importantly, local injections of dexamethasone have achieved good outcomes in clinical practice. In our prospective small-scale case study, which included 14 cases, 11 cases achieved drug-independent remission; 1 case achieved drug-dependent remission, 1 case improved; 1 case failed to improve. The overall cure rate was 85.7%. Although the mechanism of local hormone therapy for OMG remain unclear, the clinical effect cannot be denied.

Among numerous studies have investigated oral drug therapy for OMG, few have examined strabismus, ptosis, or quantified measurements of the range of eye movement as outcomes [6, 18, 19]. Kupersmith et al. [18] reported on the use of prednisone and pyridostigmine for the treatment of patients with OMG. One month later after treatment, the degree of strabismus had not changed significantly in the pyridostigmine group and had decreased from 12.2 PD to 5.5 PD in the prednisone group. Among 51 patients who responded to prednisone treatment, 26 experienced symptom recurrence as the dose of prednisone was decreased. At 2 years of follow-up, only 12 of 55 patients in the prednisone group reported that symptoms were completely relieved. No patient in the pyridostigmine group was completely relieved. Park et al. [19] followed 20 patients with MG who had obvious paralytic strabismus as the initial symptom for 45.4 ± 39.7 months. After systemic drug therapy (hormone and cholinesterase inhibitor), only 6 patients (21.4 ± 11.1) had responded well to treatment and reported the disappearance of diplopia. Four patients (14.3%) had partial reactions, and 18 cases (64.3%) had slight or no response to treatment; 10 cases (35.7%) continued to suffer from strabismus of > 15 PD. Six cases underwent strabismus surgery. Vanikieti et al. [6] performed a retrospective study of the effect of oral prednisone and/or pyridostigmine for OMG in children. Only 13 (21.67%) of 60 children with blepharoptosis achieved drug-independent remission, and only 3 (7.9%) of 38 patients with ophthalmoplegia achieved drug-independent remission. About 50% of patients responded slightly or not at all to oral medication. We summary of the results of this study and previous reports of oral hormonal therapy for OMG in Table 3.In contrast to those previous reports on the use of oral drugs, our study show that local injection of dexamethasone has better treatment result for OMG.

**Table 3:** Summary of the results of this study and previous reports of oral hormonal therapy for OMG

Author	Drug and treatment objects	Follow-up Time	Number of cases	Complete remission rate at the end of follow-up
Mark J. Kupersmith [16]	Prednisone for OMG	2 years	55	12 patients(21.8%)
Kyung-Ah Park [17]	prednisone and/or azathiorpine for Initial MG with diplopia	45.4 ± 39.7 months	28	6 patients (21.4%)
Kavin Vanikieti [6]	prednisone and/or pyridostigmine for juvenile OMG	95 months	62	Blepharoptosis:13 of 60 (21.67%); ophthalmoplegia:3of 38 (7.9%) .
Our study	Dexamethasone peribulbar or extraocular muscle injection for OMG	16.0 ± 6.33 months	14	11 patients(78.5%)

In our study, we preferred directly inject the drug into the affected extraocular muscles, typically the medial rectus, and investigated the duration of remission for various symptoms. We believe that this approach may help to identify the optimal therapeutic approach with which to accelerate recovery from extraocular paralysis. Among the patients who ultimately recovered from ptosis, the time to recovery time was ≤1 week. The average time to recovery from diplopia was 1.75 ± 0.62 weeks; the average time to recovery of ocular duction was 4.55 ± 2.44 weeks. The recovery of extraocular paralysis is still the most difficult, which may take several months. We found that the recovery of extraocular paralysis is the most difficult, which may take several months, and the recovery time is related to the degree of muscle paralysis. The recovery time is related to the degree of muscle paralysis. Compared with systemic steroids, local dexamethasone injections have a faster onset in OMG patients. Oral steroid-induced clinical improvement usually begins within 2 to 4 weeks, with marked improvement requiring 6–8 weeks [20].

Notably, although the direct injection of extraocular muscles was chosen, the sequence of symptom recovery observed in patients of our study was similar to that observed in patients who received oral drug therapy [6, 18, 19, 21].we also noted the neck muscle farthest from the injection point recovered fastest. We sought to identify the reason for this phenomenon. Nan et al. [22] reported that a single sub-Tenon injection of triamcinolone acetonide (TA) was able to diffuse throughout the globe, including the aqueous, iris-ciliary body, vitreous, neuroretina, retinal pigment epithelium, and choroid, with therapeutic concentrations maintained for at least 30 days. Roesel et al. [23] observed similar results for the use of periocular corticosteroid injections, administered via the sub-Tenon route or as an orbital floor injection, for the treatment of uveitis. Based on the above research, we speculate that periocular corticosteroid injections could easily diffuse into adjacent muscle tissues and thus reach therapeutic concentrations. Recent studies have reported that high-dose intravenous methylprednisolone therapy in patients with OMG [24] or mild GMG [25] achieves faster improvement with better efficacy than oral prednisone. Dexamethasone injection into the periocular or extraocular muscles can also reach other parts of the body through circulating blood. This capacity for diffusion may explain the rapid relief of neck-related symptoms observed in this study. Sub-Tenon injections may achieve the same treatment effects as extraocular muscle injections while reducing risk for muscle hemorrhage and local injury.

Although most of the patients had good results, we noticed that two elderly patients with severe blepharoptosis had poor results (Patients 7 and 13). In addition to the fact that some patients are not sensitive to hormones, we also note that the results of systemic oral medication therapy show that the treatment of OMG in children seems to be better than that in older people [6]. Whether the same problem exists in local therapy remains to be seen further.

For OMG that is treated with systemic medication, continued corticosteroid therapy is typically necessary to prevent recurrence and conversion to GMG. Considering the side effects of hormones, we did not administer continuous injection therapy to patients whose symptoms had resolved. In this study, 10 of 14 patients (71.3%) remained stable during the follow-up period after the treatment was stopped. Some studies suggest that patients who receive immune interval therapy during the early stage of OMG are less likely to develop systemic disease [18, 26–29]. Agius [16] reported that GMG may be due to increased antibody production and the expansion of antigenic molecular targets. The duration of the initial phase of immune attack on the neuromuscular junction may be an important determinant of disease severity. We speculate that the effectiveness of topical corticosteroids in alleviating OMG symptoms may reduce the possibility of recurrence and aggravation. The dramatic results of this study may also reflect a statistical error caused by the short observation time and the small number of cases. In conclusion, the long-term effects of local hormone therapy on OMG require further study.

This study had some limitations. Firstly, the mechanism of local hormone injection in the treatment of OMG is not clear. Secondly, there is no clinical comparison with systemic drugs. In addition, the number of cases included in this study is small. As a systemic immune disease, MG commonly persist over time, despite temporary remission. Therefore, further studies will be necessary to determine the optimal site to be used for injection, the optimal time interval between injections and the optimal course of treatment. The stability of the curative effect achieved, the recurrence rate, and the rate of conversion will need to investigated on a larger scale and for a longer period of time.

## Conclusion

In our small, prospective study, the local injection of dexamethasone in the treatment of OMG achieved good short-term results, supporting its use as a partial replacement for oral drug therapy. However, its mechanism still needs to be explored, and larger clinical controlled studies are also needed.

## Supplementary information

Additional file 1: **Table S1.** The Summary of patient characteristics and injection sites. (DOCX 13 kb)

## Data Availability

The data are available from the corresponding author upon reasonable request.

## Abbreviations

MG: Myasthenia gravis
OMG: Ocular myasthenia gravis
GMG: General myasthenia gravis
JIA: Juvenile idiopathic arthritis
CT: Computed tomography
AChR-Ab: Acetylcholine receptor antibody
MRI: Magnetic resonance imaging
PD: Prism diopters
SE: Spherical equivalents
BCVA: Best-corrected visual acuity
OD: Right eye
OS: Left eye
RMR: Right medial rectus muscle
LMR: Left medial rectus muscle
BMR: Bilateral medial rectus muscles
LSR: Left superior rectus muscle
RP: Right peribulbar area
RLR: Right lateral rectus muscle
