# Supplementary material for: Local use of dexamethasone in the treatment of ocular myasthenia gravis
Source: BMC Ophthalmol. 2020 Oct 28;20:432. doi: 10.1186/s12886-020-01697-2 (PMC7594471; doi:10.1186/s12886-020-01697-2)
Supplement: Supplementary file 1 — Table S1. The Summary of patient characteristics and injection sites. (DOCX 13 kb) [file 12886_2020_1697_MOESM1_ESM.docx]

Supplemental Table 1.

| **Patient No.** | **Patient Characteristics** | **Injection Site** |
| --- | --- | --- |
| **1** | Unilateral adduction limitation, mild/moderate blepharoptosis | Unilateral medial rectus |
| **2** | Unilateral adduction limitation, mild/moderate blepharoptosis | Unilateral medial rectus |
| **3** | Unilateral adduction limitation, mild/moderate blepharoptosis | Unilateral medial rectus |
| **4** | Exotropia, no duction limitation | Bilateral medial rectus |
| **5** | Unilateral adduction limitation, mild/moderate blepharoptosis | Unilateral medial rectus |
| **6** | Unilateral limitation in adduction and supraduction, mild/moderate blepharoptosis | Unilateral medial rectus |
| **7** | Limitation of left ocular adduction and supraduction, severe left eyelid droop | Unilateral medial rectus and superior rectus injection |
| **8** | Unilateral adduction limitation, mild/moderate blepharoptosis | Unilateral medial rectus |
| **9** | Exotropia, no duction limitation | Bilateral medial rectus |
| **10** | Unilateral adduction limitation, mild/moderate blepharoptosis | Unilateral medial rectus |
| **11** | Unilateral limitation in adduction and supraduction, mild/moderate blepharoptosis | Unilateral medial rectus |
| **12** | Unilateral adduction limitation, mild/moderate blepharoptosis | Unilateral medial rectus |
| **13** | No duction limitation, severe right ptosis, mild exotropia | Unilateral Peribulbar |
| **14** | Unilateral abduction limitation, mild/moderate blepharoptosis | Unilateral medial rectus; unilateral peribulbar |
